# Utilization of Vicryl Bridging Mesh in Orthotopic Liver Transplantation to Achieve Tension-Free Abdominal Wall Closure: A Case Series

**DOI:** 10.1155/2020/4716415

**Published:** 2020-03-19

**Authors:** Ea-sle Chang, Alice Martino, Adam Welu, Mustafa Nazzal

**Affiliations:** Saint Louis University, Department of Surgery, Saint Louis, MO 63104, USA

## Abstract

Difficulty in primary fascial closure of the abdomen in transplant patients is a common challenge. Abdominal wall tension may have detrimental effects on the newly transplanted graft due to compression, and blood flow hindrance, potentially leading to ischemia or thrombosis and possibly graft failure. Furthermore, patients will be at risk of developing fascial ischemia and dehiscence. Myocutaneous flaps, temporary closure with silastic mesh, abdominal wall transplants, and even graft reduction, bowel resection, and splenectomies have been practiced with varying degrees of success. In this study, we present four cases of patients who underwent orthotopic liver transplantation (OLT) with bridging Vicryl knitted mesh (ETHICON VKML VICRYL-Polyglactin 910-30 × 30 cm) to relieve the tension during the closure. Our results show that these patients, despite having a high average Model End-Stage Liver Disease (MELD) score of 25, had a good liver function at the time of discharge and continue to upon follow-up. They had a relatively short length of stay (LOS) in both the intensive care unit (ICU) and in the hospital, an average of 3.5 days and 9 days, respectively. Our case series successfully show that utilizing a bridging Vicryl knitted mesh is a reasonable approach to attain tension-free abdominal closure in OLT with satisfying results.

## 1. Introduction

Tension-free abdominal closure is a well-described concept for a successful postoperative course in surgery. Large-for-size liver transplants present a challenge to achieving a tension-free primary abdominal closure. Even in cases of closely matched liver size, edema of the graft or the bowel may develop after intraoperative resuscitation, portal vein clamping, and organ reperfusion, making primary tension-free closure impossible [1]. The risks of closure under tension are similar to those in the general population, notably the development of abdominal compartment syndrome and fascial ischemia and dehiscence. Orthotopic liver transplantation (OLT) patients also risk the loss of the graft due to increased abdominal pressure compressing the graft vasculature, resulting in ischemia [1]. Although an uncommon problem in the adult population, the inability to close the abdomen in a tension-free manner after OLT has potentially devastating effects on graft and patient survival.

**Figure 1:**
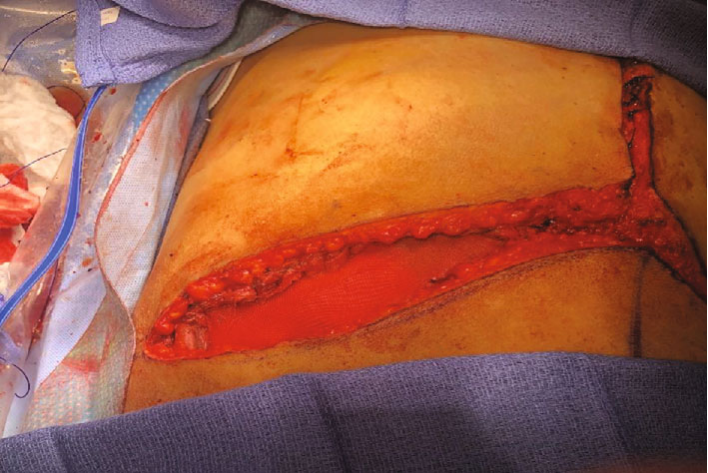
Bridging Vicryl knitted mesh inserted in the right subcostal part of the Mercedes Benz incision.

Several techniques have been described to allow tension-free abdominal closure, including myocutaneous flaps, temporary closure with silastic mesh, component separation, abdominal wall transplants, graft reduction, and even splenectomies and bowel resection. However, not all of these methods are advantageous in OLT patients. For example, component separation is another option to relieve tension but is commonly done with vertical incisions instead of the transverse incision used in OLT [2]. Myocutaneous flaps, graft reduction, splenectomy, or bowel resection all prolong an already protracted and technically demanding operation. This can increase the risks in OLT patients, who are often coagulopathic and have poor wound healing as a result of their underlying liver disease [3]. The use of mesh is a viable alternative to these options. Temporary mesh, however, necessitates another operation in the post-transplant period, and have increased potential for the development of hernias. Methods that have been proposed and utilized to overcome these factors include regional flaps, prosthetic mesh, temporary silastic mesh, and abdominal wall transplants [4–6]. Here, we present four patients at our institution that underwent abdominal closure utilizing a bridging Vicryl knitted mesh (ETHICON VKML VICRYL-Polyglactin 910-30 × 30 cm) to salvage the larger-sized grafts. At the time of the operation, it was decided that tension-free closure of the abdominal wall would be difficult due to the donor allograft size. The mesh is utilized in bridging fashion, and we typically cut the mesh into an ellipse and use 2 layers of the mesh to enforce it. Subsequently, the mesh is sutured to the external oblique muscle in the right subcostal incision area using a number 1 polydioxanone suture in a running fashion (Figure 1).

## 2. Case Series

Patient 1 is a 69-year-old female whose primary cause of the liver disease was nonalcoholic steatohepatitis complicated by hepatocellular carcinoma (HCC). Her preoperative Model End-Stage Liver Disease (MELD) score was 9 and 28 after the addition of HCC points. She also had multiple medical comorbidities including hypertension, obstructive sleep apnea, and obesity and a past surgical history of Cesarean section and hysterectomy. She received a deceased donor orthotopic liver transplant, performed with piggyback technique of veno-veno bypass with an arterial reconstruction of donor celiac artery to recipient right replaced hepatic artery and choledocho-choledochostomy. Patient 1 had a height of 154.9 cm and a weight of 84.4 kg, while the donor had a height of 170.2 cm and a weight of 122.6 kg, giving a donor-weight-to-recipient-weight ratio of 1.45. The liver had a volume of 2049 cubic centimeters (cm^3^) (as estimated by donor preoperative CT volumetric studies) and was deemed to be large relative to the patient's abdomen. At the end of the transplant, the fascia could not be closed primarily without risking compression of the graft. A Vicryl knitted mesh was used as a bridge to close the fascia without tension. She did well postoperatively and was discharged on postoperative day 6. During the course of her follow up, she developed a right anterolateral ventral hernia. The repair has been delayed due to concerns of recurrence given her BMI of 33. Her transplanted liver is doing well, with normal liver function tests at 21 months postoperatively.

Patient 2 is a 54-year-old male with hepatitis C with subsequent hepatocellular carcinoma as a primary cause of liver disease, complicated by esophageal varices, portal hypertension, and ascites requiring paracentesis. His preoperative MELD score was 17. He received a deceased donor orthotopic liver transplant with a graft volume of 1670 cm^3^ (as estimated by donor preoperative CT volumetry.) His height was 165 cm and his weight was 66.9 kg, and his donor's height and weight were 172.2 cm and 70.7 kg, respectively. His donor-weight-to-recipient-weight ratio was 1.05. The transplant was performed using a piggyback technique of veno-venous bypass with a choledocho-choledochostomy and recipient ductoplasty. Primary fascial closure was not possible due to the large liver size, so a bridging Vicryl knitted mesh was inserted in the right subcostal region to prevent abdominal compartment syndrome. On postoperative day 4, the patient's liver enzymes were elevated, requiring a transjugular liver biopsy that showed mild acute cellular rejection. This was resolved with three days of pulse steroids. The patient recovered well and was discharged on postoperative day 11. MRI performed 4 months postoperatively demonstrated a hernia in the lateral abdominal wall. The patient remains asymptomatic and does not wish to pursue repair. Graft function remains excellent at 26 months post-op.

Patient 3 is a 59-year-old female whose primary cause of the liver disease was nonalcoholic steatohepatitis with a preoperative MELD score of 29. Her liver disease was complicated by hepatic encephalopathy and ascites requiring paracentesis. Her comorbidities included depression, anxiety, colitis, and tricuspid regurgitation, and history of roux-en-y gastric bypass. She received a deceased donor orthotopic liver transplant with a graft volume of 1977 cm^3^ (as estimated by donor preoperative CT volumetry). At the time of surgery, her height was 167.6 cm, and her weight was 78.1 kg. Her donor height and weight were 165.1 cm and 92 kg, respectively, giving a donor-weight-to-recipient-weight ratio of 1.18. The transplant was performed using the piggyback technique on veno-venous bypass with choledocho-choledochostomy and recipient ductoplasty. A large amount of ascites was evacuated intraoperatively. Due to large graft size, a bridging Vicryl knitted mesh was placed in the right lateral part of the chevron incision to avoid compartment syndrome. On postoperative day 4, the level of her liver enzymes was acutely elevated. Liver ultrasound showed normal resistive indices, and she was started on stress dose steroids for suspected acute cellular rejection. Her liver enzymes responded appropriately to the steroids. Her postoperative course was also complicated by hypotension requiring pressors for 48 hours, acute kidney injury, and ileus, all of which resolved by her discharge on postoperative day 10. She is currently five months postoperatively, and her liver enzymes remain within normal limits.

Patient 4 is a 61-year-old female whose cause of the liver disease was primary biliary cirrhosis complicated by ascites not requiring paracentesis and esophageal varices, with a preoperative MELD of 26. Her medical history also included diffuse alveolar lung disorder and fibromyalgia. She received a deceased donor orthotopic liver transplant with a graft volume of 1909 cm^3^ (as estimated by donor preoperative CT volumetry) using the piggyback technique of veno-venous bypass with a choledocho-choledochostomy and portal vein eversion thrombectomy. Her height was 154.9 cm, and her weight was 56.5 kg, while her donor's height and weight were 157.5 cm and 63.3 kg, respectively, giving a donor-weight-to-recipient-weight ratio of 1.12. Due to concerns of compartment syndrome because of large graft size, a bridging mesh was utilized to achieve a tension-free fascial closure. Her postoperative course was complicated by possible acute rejection evidenced by elevated alkaline phosphatase, which responded to 3 days of stress-dose steroids. She was discharged on postoperative day 8. She is currently 8 months postoperatively and doing well with good graft function.

## 3. Results

Our case series had four patients (*n* = 4) with three females and one male. The average MELD score of our four patients was 25. The average donor weight-to-recipient ratio in our patients was 1.20. Length of stay (LOS) in the intensive care unit (ICU) and in the hospital was an average of 3.5 days and 9 days, respectively.

## 4. Discussion

Various methods of abdominal wall closure have been proposed especially in patients undergoing visceral transplantations [2, 4, 7]. Many publications have been presented with the pediatric population or with patients undergoing small bowel transplantation [4, 8–10]. Patients requiring small bowel transplants have had multiple laparotomies, with an increased likelihood of having developed entero-atmospheric fistulae, wound infections, scar tissue, and contractures; moreover, most of these patients have lost a large volume of their intestinal mass and thus have smaller abdominal cavity [9]. This difficulty is encountered in other visceral organ transplantation, as well, specifically in the pediatric population, due to the size discrepancy between the donor and the recipient. This problem, though not as prevalent, also exists in the adult population. Reasons for difficulty in primary fascial closure in orthotopic liver transplantation (OLT) include donor-recipient size mismatch, post-reperfusion hepatic edema, coagulopathy, or intestinal edema with portal vein clamping [1, 4, 9]. Due to extensive hemorrhage encountered in transplant, patients undergo massive intraoperative fluid resuscitation in the operating room, predisposing the graft, and recipient bowel, to edema [5, 11].

The concept of tension-free closure is well-known in the surgical world for successful repairs and healing [4, 5]. This is especially crucial in abdominal wall closure after transplant as compression of the organs will compromise the vasculature of the graft, which may lead to ischemia or thrombosis and ultimately cause to fail. The tension may also impede the recipient's respiration and increase their central venous pressure, decreased urine output, compromise the blood supply to the abdominal wall, predisposing them to entero-atmospheric fistula formation, herniate and possibly eviscerate [5, 6]. The insertion of the mesh in the right subcostal part of the incision serves to reduce the tension on the hilum of the liver and thus prevent any kinking in the hilar vasculature; moreover, the bulkiest part of the liver allograft is the right lobe, and therefore, the right subcostal region would have the highest point of tension. An added advantage to inserting the mesh in the right subcostal region rather than any other part of the incision is the mesh will be in direct contact with the right lobe of the liver rather than the bowels and thus reducing the chance of entero-atmospheric fistula formation.

The average donor weight-to-recipient ratio in our patients was 1.20. This disparity can be compared with a study by Carlsen and colleagues. They found in their study that patients with difficulty in primary abdominal closure had a significantly higher donor weight-to-recipient weight ratio, 1.09 vs. 0.64, *p* < 0.005 [12]. Body weight may be used as a reference point to predict the size of the graft, but this may be inaccurate as the left lateral segment, segments two and three, has been shown to vary widely even amongst the donors of the same weight [10]. As such, it is truly upon the judgment of the donor surgeon to determine the feasibility of the transplant at the donor operation and then the recipient surgeon at the time of the recipient operation to determine whether tension-free closure may be possible without compromising the graft. All of the patients did well postoperatively with a relatively short length of stay (LOS) in the intensive care unit (ICU) and in the hospital, an average of 3.5 days and 9 days, respectively.

As briefly discussed earlier, there are multiple methods of achieving tension-free abdominal closure. Benedetto et al. demonstrated 3 patients that underwent myocutaneous flaps and one patient that underwent abdominal wall transplantation [6]. Although using a flap for closure avoids using foreign material for reconstruction, it subjects the patients to not only longer procedure but also permanent anatomic changes. As organ availability is difficult to predict and hence the size, planning for the flap with a plastic surgeon would be challenging. Similar obstacles of longer and more complex surgery apply to abdominal wall transplantation. With these patients, not only are the visceral organ grafts but also the abdominal wall graft is at risk of rejection, making the potential of complications even higher. In the pediatric population, temporary closure with silastic mesh has been widely utilized. This was also demonstrated in the adult population in a study done by Jafri and colleagues [5]. No significant difference in outcomes was seen in the primary closure group versus the silastic temporary closure group, except for the requirement of significantly more blood products in the temporary closure group [5]. Most of the patients were able to eventually achieve primary closure in an average of 3.4 days, ranging from 2 to 9 days, with no differences in wound complications, rate of hepatic artery thrombosis or stricture, portal vein thrombosis or stricture, biliary complications and allograft, and patient survival [5]. Despite the success of the eventual closure of the abdominal wall in these patients, however, applying the temporary silastic mesh subjected these patients to undergo another surgery during the crucial postoperative period. Consequently, their length of stay in the hospital was prolonged. Soin et al. proposed the method of using a bridging polypropylene mesh with the subsequent reduction in stages until removal of the mesh [10]. This again, however, predisposes the patients to the risk of undergoing multiple surgeries along with the increased risk of developing adhesions and scar tissues, moreover compromising graft function and leading to further complications in very sick recipients, which would translate to higher morbidity and mortality and longer hospital stay. This method, in the adult population, may be unnecessary as the size discrepancy is not as drastic.

Other methods to compensate for the loss of domain have included graft reduction, splenectomy, bowel resection, complete adhesiolysis to maximize intraperitoneal space, and optimizing fluid resuscitation by utilizing colloid fluids [12]. Needless to say, the reduction of graft by performing partial hepatectomy makes the operation much more difficult and predisposes to increased complications. Not all institutions that perform transplants will perform split liver transplantation. Splenectomy may provide more space, but studies have shown that these patients have done poorly, including death from post-transplant sepsis [12, 13]. Component separation is a widely accepted method of primarily closing complex abdominal walls or patients with loss of domain. This method, however, is very difficult to achieve in liver transplants due to the transverse orientation nature of the incision, instead of midline and vertical.

As discussed, the aforementioned methods of achieving tension-free abdominal closures have advantages and disadvantages. We elected for a bridging Vicryl mesh at the time of the surgery for our patients. Our patient population had not only multiple comorbidities but also a high MELD score. The risk of patients having postoperative complications is increased with increased MELD, and the average MELD score of our four patients was 25. The patients are generally frail, debilitated, and ill at the time of surgery. Our goal was to perform a safe surgery to salvage and optimize graft function and minimize the operation time and recovery time for these patients. We were able to achieve these goals, as represented by the short LOS in the ICU and in the hospital. The average operation times for these patients were also approximately 8.7 hours. All of these patients, at current follow-up, have excellent graft function with 0% mortality. Moreover, utilizing a Vicryl mesh is advantageous as re-exploration in the immediate postoperative period would be easily achieved, and replacing the Vicryl mesh is technically feasible and cost-effective.

Due to the well-known risks of closing the fascia under tension, we elected to use a bridging Vicryl mesh. Although two patients in our case series developed incisional hernias, their overall recovery and improved clinical status due to functioning graft would allow a safe and more controlled condition for an elective incisional hernia repair in the future. There was no need for reoperations in any of our patients which expedited recovery and shortened hospital stay.

The two patients in our group have not undergone repair for distinct reasons. Patient 1 has a high BMI of 33 that will drastically increase her likelihood of recurrence despite repair. Patient 2, on the other hand, has no adverse effects from the hernia and has chosen the nonoperative management. The justification for Vicryl mesh as our choice for reconstruction is based on the fact that it is an absorbable mesh. By incorporating a mesh that will eventually be absorbed, the risk of infection due to a foreign object in the body is minimized. Even though absorbable mesh may have a higher incidence of developing hernia when used as a bridging mesh, if and when repairs are pursued in the future, this will be technically less challenging. Moreover, we always inserted the mesh in the right subcostal region lying directly on the liver surface so that there would be no contact between the mesh and other viscera preventing any fistula formation. Furthermore, utilizing a permanent mesh in immunocompromised transplant patients will have a potential increased risk of infection, which may be detrimental in the outcome. They are also at higher risk of erosion into abdominal viscera, adhesions, fistula formation, and infections due to the nonabsorbable nature [14].

The incidence of incisional hernia after OLT has been reported to range between 1% and 32.4% [14–16]. The trend of the incidence has been uprising, possibly due to longer survival and follow-up and due to a larger patient population with high MELD undergoing surgery. In spite of the belief that immunosuppression predisposes patients to develop an incisional hernia in OLT, the more contributive risk factors were same as for other major abdominal surgeries, including diabetes, age > 45 years, male sex, smoking history, chronic obstructive pulmonary disease (COPD), malnutrition, previous abdominal surgeries, and obesity [15, 16]. The presence and the extent of ascites were significant risk factors present in end-stage liver disease patients that significantly increased the risk of developing a hernia [15, 16]. Three of the four patients had ascites at the time of transplant. Moreover, our patients possessed a number of the risk factors that were listed to predispose them to develop hernias, including but not limited to age > 45 years, COPD, malnutrition, and obesity. Three of our four patients also underwent additional high-stress dose steroids due to concern for rejection. This additional exposure to steroids further increased their chance of developing a hernia. The adverse effects of steroids in delaying healing and decreasing the strength of tissue are well-known.

We have demonstrated four patients that have safely undergone OLT despite concerns of donor-recipient size mismatch with a satisfactory postoperative course with the utilization of Vicryl bridging mesh. Even with higher MELD score and multiple comorbidities, their LOS in the ICU and the hospital was short. Also, all of the grafts are functioning well at most recent follow-up appointments. All patients underwent OLT with a piggyback technique despite the large size of the graft without any signs of caval compression by the large graft. In our experience, Vicryl bridging mesh can serve as an effective and safe tool for preventing graft compromise in recipients that are at risk of abdominal closure with tension at the time of transplant.

## 5. Conclusion

Multiple methods of achieving a tension-free abdominal wall have been proposed in the literature in transplant candidates. The experience at our institution shows that using Vicryl bridging mesh is a safe and acceptable method to prevent abdominal compartment syndrome in these patients, without the need for multiple immediate operative interventions and with excellent graft and patient survival.

## Abbreviations

MELD: Model end-stage liver disease
LOS: Length of stay
ICU: Intensive care unit
OLT: Orthotopic liver transplantation
HCC: Hepatocellular carcinoma.
